# ReefMedMol: Mollusca from the infralittoral rocky shores - the biocoenosis of photophilic algae - in the Mediterranean Sea.

**DOI:** 10.3897/BDJ.4.e7516

**Published:** 2016-07-06

**Authors:** Dimitris Poursanidis, Drosos Koutsoubas, Christos Arvanitidis, Giorgos Chatzigeorgiou

**Affiliations:** ‡ University of the Aegean, Dept. of Marine Science, Mytiline, Lesvos, Greece; § Foundation of Research and Technology - Hellas, Heraklion, Crete, Greece; | Hellenic Centre for Marine Research, Heraklion, Crete, Greece

**Keywords:** Infralittoral zone, Mollusca, Reefs, 1170, Photophilic algae, Mediterranean Sea, Biodiversity, 1969–2014

## Abstract

**Background:**

This paper describes two datasets on the molluscan fauna from the Mediterranean infralittoral reef ecosystem - the biocoenosis of photophilic algae. The ﬁrst dataset is taken from the East Mediterranean node of the NAGISA project. The second one is a compilation based on the available published material in peer - reviewed journals as well as from the accessible grey literature. These datasets cover a time period of 43 years from 1969 to 2012 from several locations spanning the Mediterranean Sea.

**New information:**

This dataset is the only one available from this important Mediterranean Habitat, coded as 1170 in the Habitats Directive (92/43/EEC) and can provide valuable information on the needs of ecosystems functions and services assessment, habitat and species conservation as well as marine spatial planning.

## Introduction

The Mediterranean Sea is the largest enclosed marine environment globally (Coll et al. 2010). A large proportion of its coastline is rocky and mainly made by limestone, a defining characteristic of this marine basin (Gerovasileiou and Voultsiadou 2012). There, marine hard substrates exists which is of great ecological and economic value due to the high structural complexity on which rich marine communities are based. Yet, several goods and services are provided by (Salomidi et al. 2012). Due to the importance of this marine ecosystem, Habitat Directive 92/43/EEC includes it under the code 1170 (Reefs). In the infralittoral zone of the hard substrate, several biocoenoses exist (Pérès and Picard 1964). Among them, the biocoenosis of the photophilic algae is the one that dominates the hard substrates in depths up to 30 meters, depending on the water turbidity, light penetration and availability of nutrients (Coppejans 1980). Macroalgae, the main component of this biocoenosis, provides an excellent host for food and shelter to the taxonomic group of molluscs. This is one of the most important animal groups found in the Mediterranean Sea, and its molluscan fauna is the best known in the world (Sabelli and Taviani 2013) while from the 17,000 marine species that have been found in the Mediterranean, 13% are molluscs (Coll et al. 2010). The biocoenosis of photophilic algae is, however, poorly studied in terms of molluscan biodiversity; few studies examine the species communities in the infralittoral zone. All of them are paper-based, while the detection of them as well as the compilation of the included published information about the species and other associated data (sampling method, depths, location, time period) cannot be retrieved automatically by machine-learning methods and tools.

This study attempts to expand the current knowledge on the rocky infralittoral zone of the Mediterranean Sea by providing occurrence data of molluscan species from two diﬀerent sources. The ﬁrst one comes from the sampling campaign of the NAGISA project in the East Mediterranean Sea while the second one comes from the compilation of the available and accessible published material in peer-reviewed journals and grey literature. The present datasets include georeferenced and fully documented information from 22 regions/sampling sites across the Mediterranean Sea, from 1969 to 2012 (Table 1) on 599 species of molluscs (Table 2). Additional information based on the dataset of the NAGISA project about the recorded individuals per species is alsoincluded (127 species, 10326 individuals).

## Project description

### Title

This dataset combines the data of two sources: (a) the monitoring of infralittoral rocky shores in Crete in the framework of the NaGISA project (Natural Geography in Shore Areas, http://www.coml.org/projects/natural-geography-shore-areas-nagisa); (b) the collection and indexing of available and accessible paper published material in peer-review journals as well as grey literature sources.

### Personnel

Christos Arvanitidis, HCMR (NaGISA project coordinator, sample collection), Dimitris Poursanidis, FORTH & UniAegean (sample collection, species identification, data management, literature collection, information indexing), Georgios Chatzigeorgiou, HCMR (sample collection), Dimitra Mavraki (data management), Drosos Koutsoubas, UniAegean (species identification).

### Study area description

**Figure 1.**
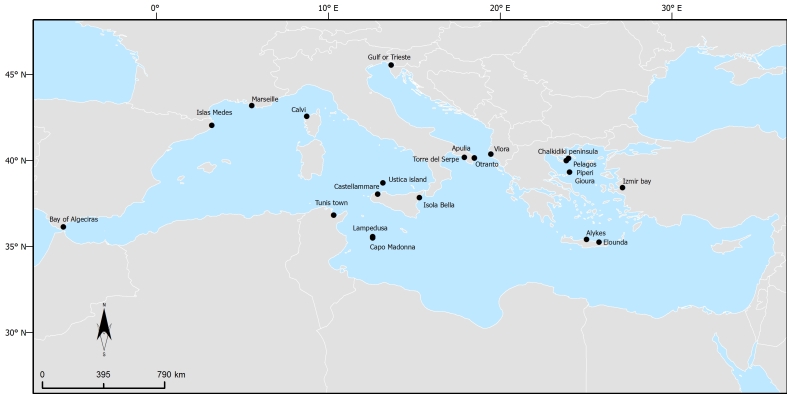
Мap of the locations.

This dataset includes records from 19 sampling sites at 18 different locations (Table 1,Fig. 1).

**East Mediterranean NAGISA project data - Alykes and Elounda**: Both sampling sites are located on the North coast of Crete (Eastern Mediterranean) and are characterised by a continuous hard bottom habitat with dense algal coverage (*Cystoseira* spp., *Sargassum* sp., *Corallinales* spp.) and a moderate wave exposure. The area of Alykes has on average a denser algal coverage than the area of Elounda. The substrate is dominated by limestone rocks. Neither of the two sites is impacted by detectable anthropogenic activity, though a sandy beach in ca 500 m distance of the sampling area in Elounda is subjected to moderate beach tourism and increased leisure boat traffic in the summer months.

**Mediterranean literature data**: The majority of the information come from the analysis of the collected literature (Antit and Azzouna. 2012, Antit M. et al. 2013, Antoniadou and Chintiroglou 2007, Antoniadou et al. 2006, Antoniadou et al. 2005, Badalamenti et al. 2002, Bellan-Santini 1969, Huelin and J. 1984, Kasemi et al. 2008, Kocataş 1978, Milazzo et al. 2000, Miloslavich et al. 2013, Pitacco et al. 2014, Poulicek 1985, Sanchez-Moyano et al. 2000, Simboura et al. 1995, Terlizzi et al. 2005, Terlizzi et al. 2003, Trono 2006, Villari 1993, Russo 1998, Chemello and Milazzo 2002). The extracted information (species list per site/paper) has been indexed in a descriptive database system (Microsoft Access 2010) in order to handle and manage the collected information (meta-analysis, quering). When authors provide species identification in .sp level, this has been kept as is but without authority, as for .sp is not valid.

## Sampling methods

### Study extent

The data covers 2 sampling events under the NaGISA initiative and several independent sampling events over a time period of 45 years (1969–2012). The dataset originates from 18 sampling sites in several countries of the Mediterranean Sea. Samples were collected from the infralittoral zone from a maximum depth of 40 m (in few studies), having a maximum sampling depth of 20m. Concerning the distribution of molluscs, this habitat is understudied in the Mediterranean Sea — in fact, the Ocean Biogeographic Information System contains only 2 datasets regarding the mollusca fauna over hard bottoms for the entire Mediterranean Sea, and neither of these two are from the infralittoral zone. The present dataset thus provides an important addition to the exiting data for this habitat in the region.

### Sampling description

Samples from Crete were collected from two sites, Alykes and Elounda. Both sites were sampled in September 2007 and June 2008. Samples were collected according to the NaGISA protocol (Iken and Konar 2003). At each site, 5 sampling depths have been defined (1, 5, 10, 15, 20 m.) and five random replicate units per ddepth were collected (Chatzigeorgiou et al. 2012). In the laboratory, all samples were identified in the most precise taxonomic level possible, using the most recent literature for the taxon. Animals with half of the size of known adult size are considered as juveniles.

Samples form the other sites have been collected in different periods over the year or over several years under different frameworks. The published available information contains the sampling methods and only the species list that have consequently found in the studied area.

### Quality control

All scientific names were standardized against the World Register of Marine species using the Taxon Match tool (http://www.marinespecies.org/aphia.php?p=match). If recent taxonomic reviews were available that had not been incorporated into WoRMS at the time of standardization, nomenclature follows those reviews.

## Geographic coverage

### Description

Samples were collected at 22 regions/sampling sites across the Mediterranean Sea LME (http://www.marineregions.org/gazetteer.php?p=details&id=1905), from a maximum depth of 40 m (Table 1,Fig. 1).All data are collected from the infralittoral zone - the biocoenosis of photophilic algae.

The present dataset contains the first electronically available quantitative data on the infralittoral molluscs from the Mediterranean Sea. Moreover, it provides the first qualitative information (species list) about the molluscs from the hard substrates – the biocoenosis of photophilic algae - from that region. As for the coralligenous formations of the infralittoral zone, available dataset can be found at http://doi.pangaea.de/10.1594/PANGAEA.847623 (Poursanidis and Koutsoubas 2015).

### Coordinates

36.2173 and -6.0327 Latitude; 30.2639 and 45.7833 Longitude.

## Taxonomic coverage

### Description

The present dataset, after updating the taxonomy, contains distribution records for 599 species (Table 2).

In total, 155 families have been found over the studied biocoenosis. The distribution of species over them is quite unequal; 71 families are represented by only 1 species (singletones or rare) while only 14 families are represented by more than 10 species. Families Pyramidellidae and Rissoidae have the largest number of species, 38 and 56 respectively. Detailed information on the structure of species/families can be found in Suppl. material 1

**Figure 2.**
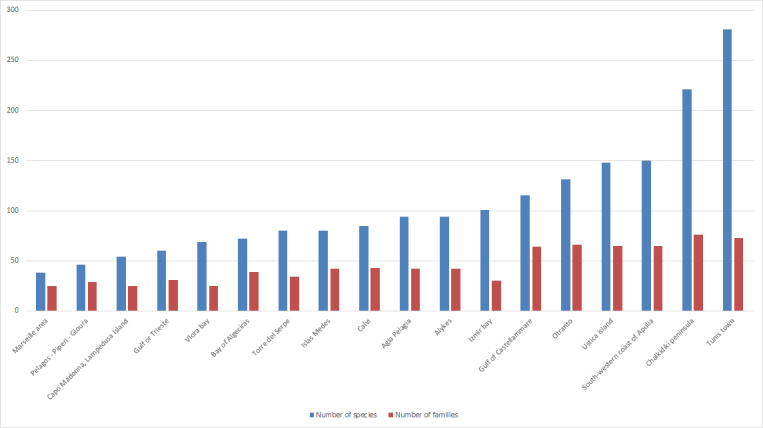
Number of species and families per sampling location. Diagram based on the data from Suppl. material 2

Species richness at the different sites is also unequal. Marseille area has the lowest number of species (38 species) while Tunis town shows the largest number of species (281 species). In terms of families, Marseille, Capo Madonna and Vlora bay have the lowest number (25 families) while Tunis town and Chalkidiki peninsula have the largest number (73 & 76 families respectively (Fig. 2)

A comparison with the Mediterranean malacofauna (excluding cephalopods) (Coll et al. 2010), which has estimated around 2000 species, shows that more than one quarter of the malacofauna (599 species) is present in the studied habitat - the photophilic algae biocoensis . Similar is the state of the malacofauna from the coralligenous formations (Poursanidis and Koutsoubas 2015) where the same percentage of molluscs have been found.

During the last decade, research in several areas in the Mediterranean has shed light on the ecology of these habitats and revealed a vast amount of information on the biodiversity of the biocoenosis of the photophilic algae (Antit and Azzouna. 2012, Antit M. et al. 2013, Antoniadou and Chintiroglou 2007, Antoniadou et al. 2006, Antoniadou et al. 2005, Badalamenti et al. 2002, Chemello and Milazzo 2002, Kasemi et al. 2008, Milazzo et al. 2000, Miloslavich et al. 2013, Pitacco et al. 2014, Sanchez-Moyano et al. 2000, Terlizzi et al. 2005, Terlizzi et al. 2003). The increased research effort on these habitats along with the initiative of the NAGISA project, addresses the need for a better understanding of the role of this important in the coastal marine environment which is under continuus pressures and threats (EEA 2015). Moreover, species list per habitat for the European waters are a request from RAC/SPA as well as from the forthcoming Integrated Monitoring and Assessment Programme (IMAP) and the Ecosystem Approach (EcAp) of UNEP/MAP and RAC/SPA (http://www.rac-spa.org/ecap).

### Taxa included

**Table:** 

Rank	Scientific Name	
kingdom	Animalia	
phylum	Mollusca	
class	Bivalvia	
class	Gastropoda	
class	Cephalopoda	
class	Polyplacophora	
class	Scaphopoda	

## Temporal coverage

**Data range:** 1969 1 01 – 2012 12 31.

## Usage rights

### Use license

Open Data Commons Public Domain Dedication and License (PDDL)

### IP rights notes

The dataset can be freely used provided it is cited.

## Data resources

### Data package title

Mollusca from the infralittoral rocky shores - the biocoenosis of photophilic algae - in the Mediterranean Sea

### Number of data sets

2

### Data set 1.

#### Data set name

Molluscs from two rocky shores of the North coast of Crete

#### Data format

Darwin Core Archive

#### Number of columns

39

#### Character set

UTF-8

#### Download URL

http://lifewww-00.her.hcmr.gr:8080/medobis/resource.do?r=molluscs_nagisa

#### Data format version

ver 1.0

#### Description

The dataset is available via the Lifewatch node of Greece of the Hellenic Center of Marine Research. The data will also be harvested by and made available through the International OBIS database, as well as through the data portal of the Global Biodiversity Information Facility (GBIF). The dataset is available as a DarwinCoreArchive, all fields are mapped to DarwinCore terms (http://rs.tdwg.org/dwc/).

This publication refers to the most recent version of the dataset available through the IPT server. Future changes to the dataset due to quality control activities might change its content or structure.

**Data set 1.** 

Column label	Column description
id	A unique identifier for the record within the data set or collection.
type	The type of the source
language	The used language
dataset	The number of the dataset
institutionCode	The name (or acronym) in use by the institution having custody of the object(s) or information referred to in the record.
collectionCode	The code of the Collection, in which belongs to
basisOfRecord	The specific nature of the data record, as described in http://rs.tdwg.org/dwc/terms/type-vocabulary/index.htm.
occurrenceID	The replicate in which the record belongs to
catalogNumber	The catalog number of the dataset
individualCount	The abundance of the specimen in the replicate
samplingProtocol	The description of the method or protocol used for sample collection.
samplingEffort	The description of the sampling effort for the specimens collection
eventDate	The exact date of the sampling event
fieldNumber	The combination of the location and the sampling year
locationID	The code of the location
higherGeographyID	The code of the higher geography
higherGeography	The marine basin that the sampling area belongs to
waterBody	The name of the water body in which the sampling location occurs.
island	The name of the island on or near which the sampling location occurs.
country	The name of the country on which the sampling location occurs.
municipality	The full, unabbreviated name of the next smaller administrative region than county (city, municipality, etc.) in which the sampling location occurs.
locality	The exact locality where the sampling procedure took place
minimumDepthInMeters	The lesser depth of a range of depth below the local surface, in meters.
maximumDepthInMeters	The greater depth of a range of depth below the local surface, in meters.
locationRemarks	The method that the location has been located by using Google Earth app.
verbatimLatitude	The geographic latitude (in decimal degrees, using the spatial reference system given in geodeticDatum) of the geographic center of a Location. Positive values are north of the Equator, negative values are south of it. Legal values lie between -90 and 90, inclusive.
verbatimLongitude	The geographic longitude (in decimal degrees, using the spatial reference system given in geodeticDatum) of the geographic center of a Location. Positive values are east of the Greenwich Meridian, negative values are west of it. Legal values lie between -180 and 180, inclusive.
geodeticDatum	The geodetic datum of the pair of coordinates
coordinateUncertaintyInMeters	The horizontal distance (in meters) from the given decimalLatitude and decimalLongitude describing the smallest circle containing the whole of the sampling location.
scientificNameID	The Life Science Identifiers code of the scientific name
scientificName	The scientific name of the taxon
scientificNameAuthorship	The authorship information for the scientificName formatted according to the conventions of the applicable nomenclaturalCode.
Kingdom	The full scientific name of the kingdom in which the taxon is classified.
Phylum	The full scientific name of the phylum in which the taxon is classified.
Class	The full scientific name of the class in which the taxon is classified.
Order	The full scientific name of the order in which the taxon is classified.
Family	The full scientific name of the family in which the taxon is classified.
Genus	The full scientific name of the genus in which the taxon is classified.
specificEpithet	The full scientific name of the specie in which the taxon is classified.

### Data set 2.

#### Data set name

Mollusca fauna from the Mediterranean reef ecosystem (1170) – the zone of the photophilic algae.

#### Data format

Darwin Core Archive

#### Number of columns

27

#### Character set

UTF-8

#### Download URL

http://lifewww-00.her.hcmr.gr:8080/medobis/resource.do?r=moll_poursani

#### Data format version

ver 1.0

#### Description

The dataset is available via the Lifewatch node of Greece of the Hellenic Center of Marine Research. The data will also be harvested by and made available through the International OBIS database, as well as through the data portal of the Global Biodiversity Information Facility (GBIF). The dataset is available as a DarwinCoreArchive, all fields are mapped to DarwinCore terms (http://rs.tdwg.org/dwc/).

This publication refers to the most recent version of the dataset available through the IPT server. Future changes to the dataset due to quality control activities might change its content or structure.

**Data set 2.** 

Column label	Column description
Source	The literature source used for
basisOfRecord	The specific nature of the data record, as described in http://rs.tdwg.org/dwc/terms/type-vocabulary/index.htm.
type	The type of the record
eventDate	The period of the observation
higherGeographyID	The code of the geographic area as is given by the http://www.marineregions.org/index.php
higherGeography	The name of the geographic area
waterBody	The name of the water body in which the sampling location occurs.
country	The name of the country in which the sampling location occurs.
locality	The specific location where the sample was taken.
minimumDepthInMeters	The lesser depth of a range of depth below the local surface, in meters.
maximumDepthInMeters	The greater depth of a range of depth below the local surface, in meters.
locationRemarks	Details on the exact location
verbatimLatitude	The geographic latitude (in decimal degrees, using the spatial reference system given in geodeticDatum) of the geographic center of a Location. Positive values are north of the Equator, negative values are south of it. Legal values lie between -90 and 90, inclusive.
verbatimLongitude	The geographic longitude (in decimal degrees, using the spatial reference system given in geodeticDatum) of the geographic center of a Location. Positive values are east of the Greenwich Meridian, negative values are west of it. Legal values lie between -180 and 180, inclusive.
geodeticDatum	The geodetic datum of the used coordinate system.
coordinateUncertaintyInMeters	The horizontal distance (in meters) from the given decimalLatitude and decimalLongitude describing the smallest circle containing the whole of the sampling location.
scientificNameID	The Life Science Identifiers code of the scientific name
scientificName	The scientific name of the taxon
kingdom	The full scientific name of the kingdom in which the taxon is classified.
phylum	The full scientific name of the phylum in which the taxon is classified.
class	The full scientific name of the class in which the taxon is classified.
order	The full scientific name of the orde in which the taxon is classified.
family	The full scientific name of the family in which the taxon is classified.
genus	The full scientific name of the genus in which the taxon is classified.
specificEpithet	The species epithet of the scientificName.
scientificNameAuthorship	The authorship information for the scientificName formatted according to the conventions of the applicable nomenclaturalCode.
taxonRemarks	Comments or notes about the taxon or name.

## Additional information

### Resource citation

Poursanidis D, Chatzigeorgiou G, Arvanitidis C. Mavraki D. and D. Koutsoubas (2015). Molluscs from two rocky shores (infralittoral zone) of the North coast of Crete, collected for the NaGISA project 2007-2008

Dimitris Poursanidis, Dimitra Mavraki & Drosos Koutsoubas (2015). Mollusca fauna from the Mediterranean reef ecosystem (1170) – the zone of the photophilic algae.

## Supplementary Material

Supplementary material 1 Number of Species per Family Data type: Microsoft Excel spreadsheet
Brief description: Summary of the number of species per family.
File: oo_83323.xlsx Dimitris Poursanidis

Supplementary material 2 Species and Families per studied site Data type: Microsoft Excel spreadsheet
Brief description: Overview of the number of taxa per sampling station
File: oo_83324.xlsx Dimitris Poursanidis

## Figures and Tables

**Table 1.** Coordinates, depth and sampling dates of the sampling localities

Country	Sampling site	Minimum depth (in meters)	Maximum depth (in meters)	Latitude	Longitude	Sampling period
Albania	Vlora bay	1	15	40.36343	19.473392	2004-2006
France	Marseille area	5	25	43.18859	5.546588	1969
France	Calvi	3	30	42.57192	8.747125	1980-1983
Greece	Chalkidiki peninsula	15	30	40.11722	23.984323	1997-1998
Greece	Chalkidiki peninsula	15	40	39.98843	23.858191	1997-1998
Greece	Chalkidiki peninsula	15	40	39.98843	23.858191	1998-1999
Greece	Pelagos - Piperi - Gioura	2	8	39.32063	24.054025	1995
Italy	Isola Bella	1	30	37.84993	15.300747	1990
Italy	Lampedusa	0	10	35.52305	12.59324	1990
Italy	Ionian coast of Salento			40.2732	17.791895	1992-2002
Italy	Capo Madonna, Lampedusa Island	1	6	35.4982	12.589623	1994
Italy	Gulf of Castellammare	16	22	38.03581	12.874333	1995
Italy	Ustica island	1	15	38.6938	13.178993	1996
Italy	Otranto	5	25	40.14283	18.507785	2000
Italy	South-western coast of Apulia	3	4	40.18485	17.92297	2002
Italy	Torre del Serpe	1	20	40.145	18.505	2004-2009
Slovenia	Gulf or Trieste	1	4	45.54799	13.662176	2008-2012
Spain	Medes island	1	30	42.04534	3.224491	1984
Spain	Bay of Algeciras	3	5	36.13232	-5.418617	1992-1993
Tunisia	Tunis town	1	2	36.82333	10.31931	2009-2010
Tunisia	Tunis town	3	15	36.82333	10.31931	2009-2010
Turkey	Izmir bay	1	20	38.41861	27.139167	1978

**Table 2.** The assembled species list in phylogenetic order.

Class	Family	Species	Authority
Polyplacophora	Chitonidae	*Chiton olivaceus*	Spengler, 1797
Polyplacophora	Chitonidae	*Chiton corallinus*	(Risso, 1826)
Polyplacophora	Chitonidae	*Chiton phaseolinus*	Monterosato, 1879
Polyplacophora	Leptochitonidae	*Lepidopleurus cajetanus*	(Poli, 1791)
Polyplacophora	Leptochitonidae	*Lepidopleurus* sp.	Risso, 1826
Polyplacophora	Leptochitonidae	*Leptochiton scabridus*	(Jeffreys, 1880)
Polyplacophora	Ischnochitonidae	*Ischnochiton rissoi*	(Payraudeau, 1826)
Polyplacophora	Ischnochitonidae	*Ischnochiton* sp.	
Polyplacophora	Callistoplacidae	*Callistochiton pachylasmae*	(Monterosato, 1879)
Polyplacophora	Callochitonidae	*Callochiton septemvalvis*	(Montagu, 1803)
Polyplacophora	Lepidochitonidae	*Lepidochitona* sp.	Gray, 1821
Polyplacophora	Lepidochitonidae	*Lepidochitona caprearum*	(Scacchi, 1836)
Polyplacophora	Lepidochitonidae	*Lepidochitona cinerea*	(Linnaeus, 1767)
Polyplacophora	Lepidochitonidae	*Lepidochitona furtiva*	(Monterosato, 1879)
Polyplacophora	Lepidochitonidae	*Lepidochitona monterosatoi*	Kaas & Van Belle, 1981
Polyplacophora	Acanthochitonidae	*Acanthochitona discrepans*	(Brown, 1827)
Polyplacophora	Acanthochitonidae	*Acanthochitona fascicularis*	(Linnaeus, 1767)
Gastropoda	Patellidae	*Patella caerulea*	Linnaeus, 1758
Gastropoda	Patellidae	*Patella rustica*	Linnaeus, 1758
Gastropoda	Patellidae	*Patella ulyssiponensis*	Gmelin, 1791
Gastropoda	Lottiidae	*Tectura virginea*	(O. F. Müller, 1776)
Gastropoda	Fissurellidae	*Diodora dorsata*	(Monterosato, 1878)
Gastropoda	Fissurellidae	*Diodora gibberula*	(Lamarck, 1822)
Gastropoda	Fissurellidae	*Diodora graeca*	(Linnaeus, 1758)
Gastropoda	Fissurellidae	*Diodora italica*	(Defrance, 1820)
Gastropoda	Fissurellidae	*Emarginula* sp.	
Gastropoda	Fissurellidae	*Emarginula adriatica*	O. G. Costa, 1830
Gastropoda	Fissurellidae	*Emarginula huzardii*	Payraudeau, 1826
Gastropoda	Fissurellidae	*Emarginula octaviana*	Coen, 1939
Gastropoda	Fissurellidae	*Emarginula tenera*	Locard, 1892
Gastropoda	Fissurellidae	*Fissurella nubecula*	(Linnaeus, 1758)
Gastropoda	Fissurellidae	*Puncturella noachina*	(Linnaeus, 1771)
Gastropoda	Scissurellidae	*Scissurella costata*	d'Orbigny, 1824
Gastropoda	Scissurellidae	*Sinezona cingulata*	(O. G. Costa, 1861)
Gastropoda	Anatomidae	*Anatoma crispata*	(Fleming, 1828)
Gastropoda	Haliotidae	*Haliotis tuberculata*	Linnaeus, 1758
Gastropoda	Haliotidae	*Haliotis tuberculata lamellosa*	Lamarck, 1822
Gastropoda	Trochidae	*Clanculus* sp.	
Gastropoda	Trochidae	*Clanculus corallinus*	(Gmelin, 1791)
Gastropoda	Trochidae	*Clanculus cruciatus*	(Linnaeus, 1758)
Gastropoda	Trochidae	*Clanculus jussieui*	(Payraudeau, 1826)
Gastropoda	Trochidae	*Gibbula adansonii*	(Payraudeau, 1826)
Gastropoda	Trochidae	*Gibbula adriatica*	(Philippi, 1844)
Gastropoda	Trochidae	*Gibbula albida*	(Gmelin, 1791)
Gastropoda	Trochidae	*Gibbula ardens*	(Salis Marschlins, 1793)
Gastropoda	Trochidae	*Gibbula divaricata*	(Linnaeus, 1758)
Gastropoda	Trochidae	*Gibbula drepanensis*	(Brugnone, 1873)
Gastropoda	Trochidae	*Gibbula fanulum*	(Gmelin, 1791)
Gastropoda	Trochidae	*Gibbula guttadauri*	(Philippi, 1836)
Gastropoda	Trochidae	*Gibbula leucophaea*	(Philippi, 1836)
Gastropoda	Trochidae	*Gibbula magus*	(Linnaeus, 1758)
Gastropoda	Trochidae	*Gibbula philberti*	(Récluz, 1843)
Gastropoda	Trochidae	*Gibbula racketti*	(Payraudeau, 1826)
Gastropoda	Trochidae	*Gibbula rarilineata*	(Michaud, 1829)
Gastropoda	Trochidae	*Gibbula tingitana*	Pallary, 1901
Gastropoda	Trochidae	*Gibbula turbinoides*	(Deshayes, 1835)
Gastropoda	Trochidae	*Gibbula umbilicaris*	(Linnaeus, 1758)
Gastropoda	Trochidae	*Gibbula varia*	(Linnaeus, 1758)
Gastropoda	Trochidae	*Jujubinus exasperatus*	(Pennant, 1777)
Gastropoda	Trochidae	*Jujubinus gravinae*	(Dautzenberg, 1881)
Gastropoda	Trochidae	*Jujubinus ruscurianus*	(Weinkauff, 1868)
Gastropoda	Trochidae	*Jujubinus striatus*	(Linnaeus, 1758)
Gastropoda	Trochidae	*Phorcus articulatus*	(Lamarck, 1822)
Gastropoda	Trochidae	*Phorcus turbinatus*	(Born, 1778)
Gastropoda	Calliostomatidae	*Calliostoma conulus*	(Linnaeus, 1758)
Gastropoda	Calliostomatidae	*Calliostoma gualterianum*	(Philippi, 1848)
Gastropoda	Calliostomatidae	*Calliostoma laugieri*	(Payraudeau, 1826)
Gastropoda	Calliostomatidae	*Calliostoma zizyphinum*	(Linnaeus, 1758)
Gastropoda	Turbinidae	*Bolma rugosa*	(Linnaeus, 1767)
Gastropoda	Skeneidae	*Skenea serpuloides*	(Montagu, 1808)
Gastropoda	Chilodontidae	*Danilia otaviana*	(Cantraine, 1835)
Gastropoda	Phasianellidae	*Tricolia* sp.	
Gastropoda	Phasianellidae	*Tricolia entomocheila*	Gofas, 1993
Gastropoda	Phasianellidae	*Tricolia miniata*	(Monterosato, 1884)
Gastropoda	Phasianellidae	*Tricolia pullus*	(Linnaeus, 1758)
Gastropoda	Phasianellidae	*Tricolia speciosa*	(Megerle von Mühlfeld, 1824)
Gastropoda	Phasianellidae	*Tricolia tenuis*	(Michaud, 1829)
Gastropoda	Phasianellidae	*Tricolia tingitana*	Gofas, 1982
Gastropoda	Colloniidae	*Homalopoma sanguineum*	(Linnaeus, 1758)
Gastropoda	Neritidae	*Smaragdia viridis*	(Linnaeus, 1758)
Gastropoda	Neritidae	*Theodoxus fluviatilis*	(Linnaeus, 1758)
Gastropoda	Cerithiidae	*Bittium* sp.	
Gastropoda	Cerithiidae	*Bittium lacteum*	(Philippi, 1836)
Gastropoda	Cerithiidae	*Bittium latreillii*	(Payraudeau, 1826)
Gastropoda	Cerithiidae	*Bittium nanum*	(Mayer, 1864)
Gastropoda	Cerithiidae	*Bittium reticulatum*	(da Costa, 1778)
Gastropoda	Cerithiidae	*Bittium submamillatum*	(de Rayneval & Ponzi, 1854)
Gastropoda	Cerithiidae	*Cerithium* sp.	
Gastropoda	Cerithiidae	*Cerithium lividulum*	Risso, 1826
Gastropoda	Cerithiidae	*Cerithium renovatum*	Monterosato, 1884
Gastropoda	Cerithiidae	*Cerithium vulgatum*	Bruguière, 1792
Gastropoda	Cerithiidae	*Cerithium scabridum*	Philippi, 1848
Gastropoda	Cerithiidae	*Cerithium vulgatum*	Bruguière, 1792
Gastropoda	Planaxidae	*Fossarus ambiguus*	(Linnaeus, 1758)
Gastropoda	Potamididae	*Pirenella conica*	(Blainville, 1829)
Gastropoda	Turritellidae	*Turritella communis*	Risso, 1826
Gastropoda	Turritellidae	*Turritella triplicata*	(Brocchi, 1814)
Gastropoda	Triphoridae	*Cheirodonta pallescens*	(Jeffreys, 1867)
Gastropoda	Triphoridae	*Cosmotriphora melanura*	(C. B. Adams, 1850)
Gastropoda	Triphoridae	*Marshallora adversa*	(Montagu, 1803)
Gastropoda	Triphoridae	*Metaxia metaxa*	(Delle Chiaje, 1828)
Gastropoda	Triphoridae	*Monophorus* sp.	
Gastropoda	Triphoridae	*Monophorus erythrosoma*	(Bouchet & Guillemot, 1978)
Gastropoda	Triphoridae	*Monophorus perversus*	(Linnaeus, 1758)
Gastropoda	Triphoridae	*Monophorus thiriotae*	Bouchet, 1985
Gastropoda	Triphoridae	*Similiphora similior*	(Bouchet & Guillemot, 1978)
Gastropoda	Cerithiopsidae	*Cerithiopsis* sp.	
Gastropoda	Cerithiopsidae	*Cerithiopsis atalaya*	Watson, 1885
Gastropoda	Cerithiopsidae	*Cerithiopsis barleei*	Jeffreys, 1867
Gastropoda	Cerithiopsidae	*Cerithiopsis fayalensis*	Watson, 1880
Gastropoda	Cerithiopsidae	*Cerithiopsis minima*	(Brusina, 1865)
Gastropoda	Cerithiopsidae	*Cerithiopsis scalaris*	Locard, 1892
Gastropoda	Cerithiopsidae	*Cerithiopsis tubercularis*	(Montagu, 1803)
Gastropoda	Cerithiopsidae	*Dizoniopsis bilineata*	(Hoernes, 1848)
Gastropoda	Cerithiopsidae	*Dizoniopsis coppolae*	(Aradas, 1870)
Gastropoda	Cerithiopsidae	*Krachia tiara*	(Monterosato, 1874)
Gastropoda	Cerithiopsidae	*Seila trilineata*	(Philippi, 1836)
Gastropoda	Epitoniidae	*Epitonium clathrus*	(Linnaeus, 1758)
Gastropoda	Epitoniidae	*Epitonium tenuicostatum*	(G. B. Sowerby, 1844)
Gastropoda	Epitoniidae	*Epitonium turtonis*	(Turton, 1819)
Gastropoda	Epitoniidae	*Gyroscala lamellosa*	(Lamarck, 1822)
Gastropoda	Eulimidae	*Eulima glabra*	(da Costa, 1778)
Gastropoda	Eulimidae	*Melanella lubrica*	(Monterosato, 1890)
Gastropoda	Eulimidae	*Melanella petitiana*	(Brusina, 1869)
Gastropoda	Eulimidae	*Melanella polita*	(Linnaeus, 1758)
Gastropoda	Eulimidae	*Melanella praecurta*	(Pallary, 1904)
Gastropoda	Eulimidae	*Vitreolina incurva*	(Bucquoy, Dautzenberg & Dollfus, 1883)
Gastropoda	Eulimidae	*Vitreolina philippi*	(de Rayneval & Ponzi, 1854)
Gastropoda	Littorinidae	*Echinolittorina punctata*	(Gmelin, 1791)
Gastropoda	Littorinidae	*Melarhaphe neritoides*	(Linnaeus, 1758)
Gastropoda	Skeneopsidae	*Skeneopsis planorbis*	(O. Fabricius, 1780)
Gastropoda	Cingulopsidae	*Eatonina cossurae*	(Calcara, 1841)
Gastropoda	Cingulopsidae	*Eatonina fulgida*	(Adams J., 1797)
Gastropoda	Cingulopsidae	*Tubbreva micrometrica*	(Aradas & Benoit, 1876)
Gastropoda	Rissoidae	*Alvania* sp.	
Gastropoda	Rissoidae	*Alvania aspera*	(Philippi, 1844)
Gastropoda	Rissoidae	*Alvania beanii*	(Hanley in Thorpe, 1844)
Gastropoda	Rissoidae	*Crisilla beniamina*	(Monterosato, 1884)
Gastropoda	Rissoidae	*Alvania cancellata*	(da Costa, 1778)
Gastropoda	Rissoidae	*Alvania cimex*	(Linnaeus, 1758)
Gastropoda	Rissoidae	*Alvania colossophilus*	Oberling, 1970
Gastropoda	Rissoidae	*Alvania discors*	(Allan, 1818)
Gastropoda	Rissoidae	*Alvania dorbignyi*	(Audouin, 1826)
Gastropoda	Rissoidae	*Alvania geryonia*	(Nardo, 1847)
Gastropoda	Rissoidae	*Alvania hallgassi*	Amati & Oliverio, 1985
Gastropoda	Rissoidae	*Alvania lactea*	(Michaud, 1830)
Gastropoda	Rissoidae	*Alvania lanciae*	(Calcara, 1845)
Gastropoda	Rissoidae	*Alvania lineata*	Risso, 1826
Gastropoda	Rissoidae	*Alvania mamillata*	Risso, 1826
Gastropoda	Rissoidae	*Alvania pagodula*	(Bucquoy, Dautzenberg & Dollfus, 1884)
Gastropoda	Rissoidae	*Alvania parvula*	(Jeffreys, 1884)
Gastropoda	Rissoidae	*Alvania rudis*	(Philippi, 1844)
Gastropoda	Rissoidae	*Alvania scabra*	(Philippi, 1844)
Gastropoda	Rissoidae	*Alvania simulans*	Locard, 1886
Gastropoda	Rissoidae	*Alvania spinosa*	(Monterosato, 1890)
Gastropoda	Rissoidae	*Alvania subcrenulata*	(Bucquoy, Dautzenberg & Dollfus, 1884)
Gastropoda	Rissoidae	*Alvania tenera*	(Philippi, 1844)
Gastropoda	Rissoidae	*Alvania tessellata*	Schwartz in Weinkauff, 1868
Gastropoda	Rissoidae	*Benthonella tenella*	(Jeffreys, 1869)
Gastropoda	Rissoidae	*Crisilla beniamina*	(Monterosato, 1884)
Gastropoda	Rissoidae	*Crisilla semistriata*	(Montagu, 1808)
Gastropoda	Rissoidae	*Manzonia crassa*	(Kanmacher, 1798)
Gastropoda	Rissoidae	*Peringiella elegans*	(Locard, 1892)
Gastropoda	Rissoidae	*Pusillina* sp.	
Gastropoda	Rissoidae	*Pusillina benzi*	(Aradas & Maggiore, 1844)
Gastropoda	Rissoidae	*Pusillina inconspicua*	(Alder, 1844)
Gastropoda	Rissoidae	*Pusillina lineolata*	(Michaud, 1830)
Gastropoda	Rissoidae	*Pusillina marginata*	(Michaud, 1830)
Gastropoda	Rissoidae	*Pusillina philippi*	(Aradas & Maggiore, 1844)
Gastropoda	Rissoidae	*Pusillina radiata*	(Philippi, 1836)
Gastropoda	Rissoidae	*Rissoa* sp.	
Gastropoda	Rissoidae	*Rissoa auriscalpium*	(Linnaeus, 1758)
Gastropoda	Rissoidae	*Rissoa decorata*	Philippi, 1846
Gastropoda	Rissoidae	*Rissoa guerinii*	Récluz, 1843
Gastropoda	Rissoidae	*Rissoa lia*	(Monterosato, 1884)
Gastropoda	Rissoidae	*Rissoa lilacina*	Récluz, 1843
Gastropoda	Rissoidae	*Rissoa membranacea*	(J. Adams, 1800)
Gastropoda	Rissoidae	*Rissoa monodonta*	Philippi, 1836
Gastropoda	Rissoidae	*Rissoa parva*	(da Costa, 1778)
Gastropoda	Rissoidae	*Rissoa scurra*	(Monterosato, 1917)
Gastropoda	Rissoidae	*Rissoa similis*	Scacchi, 1836
Gastropoda	Rissoidae	*Rissoa splendida*	Eichwald, 1830
Gastropoda	Rissoidae	*Rissoa variabilis*	(Von Mühlfeldt, 1824)
Gastropoda	Rissoidae	*Rissoa ventricosa*	Desmarest, 1814
Gastropoda	Rissoidae	*Rissoa violacea*	Desmarest, 1814
Gastropoda	Rissoidae	*Setia amabilis*	(Locard, 1886)
Gastropoda	Rissoidae	*Setia ambigua*	(Brugnone, 1873)
Gastropoda	Rissoidae	*Setia antipolitana*	(van der Linden & W. M. Wagner, 1987)
Gastropoda	Rissoidae	*Setia maculata*	(Monterosato, 1869)
Gastropoda	Rissoidae	*Setia turriculata*	Monterosato, 1884
Gastropoda	Anabathridae	*Nodulus contortus*	(Jeffreys, 1856)
Gastropoda	Anabathridae	*Pisinna glabrata*	(Megerle von Mühlfeld, 1824)
Gastropoda	Barleeiidae	*Barleeia unifasciata*	(Montagu, 1803)
Gastropoda	Caecidae	*Caecum antillarum*	Carpenter, 1858
Gastropoda	Caecidae	*Caecum clarkii*	Carpenter, 1859
Gastropoda	Caecidae	*Caecum subannulatum*	de Folin, 1870
Gastropoda	Caecidae	*Caecum trachea*	(Montagu, 1803)
Gastropoda	Caecidae	*Parastrophia asturiana*	de Folin, 1870
Gastropoda	Iravadiidae	*Hyala vitrea*	(Montagu, 1803)
Gastropoda	Tornidae	*Circulus striatus*	(Philippi, 1836)
Gastropoda	Truncatellidae	*Truncatella subcylindrica*	(Linnaeus, 1767)
Gastropoda	Vermetidae	*Dendropoma cristatum*	(Biondi, 1859)
Gastropoda	Vermetidae	*Thylacodes arenarius*	(Linnaeus, 1758)
Gastropoda	Vermetidae	*Thylaeodus rugulosus*	(Monterosato, 1878)
Gastropoda	Vermetidae	*Vermetus granulatus*	(Gravenhorst, 1831)
Gastropoda	Vermetidae	*Vermetus triquetrus*	Bivona-Bernardi, 1832
Gastropoda	Volvatellidae	*Ascobulla fragilis*	(Jeffreys, 1856)
Gastropoda	Strombidae	*Conomurex persicus*	(Swainson, 1821)
Gastropoda	Vanikoridae	*Megalomphalus azoneus*	(Brusina, 1865)
Gastropoda	Calyptraeidae	*Calyptraea chinensis*	(Linnaeus, 1758)
Gastropoda	Calyptraeidae	*Crepidula fornicata*	(Linnaeus, 1758)
Gastropoda	Calyptraeidae	*Crepidula moulinsii*	Michaud, 1829
Gastropoda	Calyptraeidae	*Crepidula unguiformis*	Lamarck, 1822
Gastropoda	Capulidae	*Capulus ungaricus*	(Linnaeus, 1758)
Gastropoda	Triviidae	*Erato voluta*	(Montagu, 1803)
Gastropoda	Triviidae	*Trivia mediterranea*	(Risso, 1826)
Gastropoda	Triviidae	*Trivia monacha*	(da Costa, 1778)
Gastropoda	Cypraeidae	*Erosaria spurca*	(Linnaeus, 1758)
Gastropoda	Cypraeidae	*Luria lurida*	(Linnaeus, 1758)
Gastropoda	Cypraeidae	*Zonaria pyrum*	(Gmelin, 1791)
Gastropoda	Ovulidae	*Pseudosimnia carnea*	(Poiret, 1789)
Gastropoda	Naticidae	*Euspira intricata*	(Donovan, 1804)
Gastropoda	Naticidae	*Euspira macilenta*	(Philippi, 1844)
Gastropoda	Naticidae	*Naticarius stercusmuscarum*	(Gmelin, 1791)
Gastropoda	Naticidae	*Neverita josephinia*	Risso, 1826
Gastropoda	Naticidae	*Notocochlis dillwynii*	(Payraudeau, 1826)
Gastropoda	Ranellidae	*Cabestana cutacea*	(Linnaeus, 1767)
Gastropoda	Ranellidae	*Charonia variegata*	(Lamarck, 1816)
Gastropoda	Bursidae	*Bursa scrobilator*	(Linnaeus, 1758)
Gastropoda	Muricidae	*Bolinus brandaris*	(Linnaeus, 1758)
Gastropoda	Muricidae	*Coralliophila brevis*	(Blainville, 1832)
Gastropoda	Muricidae	*Coralliophila meyendorffii*	(Calcara, 1845)
Gastropoda	Muricidae	*Hadriania craticulata*	Bucquoy, Dautzenberg & Dollfus, 1882
Gastropoda	Muricidae	*Hexaplex trunculus*	(Linnaeus, 1758)
Gastropoda	Muricidae	*Hirtomurex squamosus*	(Bivona Ant. in Bivona And., 1838)
Gastropoda	Muricidae	*Murexsul cevikeri*	(Houart, 2000)
Gastropoda	Muricidae	*Muricopsis cristata*	(Brocchi, 1814)
Gastropoda	Muricidae	*Ocenebra erinaceus*	(Linnaeus, 1758)
Gastropoda	Muricidae	*Ocinebrina* sp.	
Gastropoda	Muricidae	*Ocinebrina aciculata*	(Lamarck, 1822)
Gastropoda	Muricidae	*Ocinebrina edwardsii*	(Payraudeau, 1826)
Gastropoda	Muricidae	*Stramonita haemastoma*	(Linnaeus, 1767)
Gastropoda	Muricidae	*Trophonopsis alboranensis*	(Smriglio, Mariottini & Bonfitto, 1997)
Gastropoda	Muricidae	*Trophonopsis muricata*	(Montagu, 1803)
Gastropoda	Marginellidae	*Granulina boucheti*	Gofas, 1992
Gastropoda	Marginellidae	*Granulina clandestina*	(Brocchi, 1814)
Gastropoda	Marginellidae	*Granulina marginata*	(Bivona, 1832)
Gastropoda	Marginellidae	*Volvarina mitrella*	(Risso, 1826)
Gastropoda	Cystiscidae	*Gibberula caelata*	(Monterosato, 1877)
Gastropoda	Cystiscidae	*Gibberula jansseni*	van Aartsen, Menkhorst & Gittenberger, 1984
Gastropoda	Cystiscidae	*Gibberula miliaria*	(Linnaeus, 1758)
Gastropoda	Cystiscidae	*Gibberula philippii*	(Monterosato, 1878)
Gastropoda	Cystiscidae	*Gibberula recondita*	Monterosato, 1884
Gastropoda	Mitridae	*Mitra cornicula*	(Linnaeus, 1758)
Gastropoda	Costellariidae	*Vexillum ebenus*	(Lamarck, 1811)
Gastropoda	Costellariidae	*Vexillum granum*	(Forbes, 1844)
Gastropoda	Costellariidae	*Vexillum savignyi*	(Payraudeau, 1826)
Gastropoda	Costellariidae	*Vexillum tricolor*	(Gmelin, 1791)
Gastropoda	Buccinidae	*Euthria cornea*	(Linnaeus, 1758)
Gastropoda	Buccinidae	*Chauvetia affinis*	(Monterosato, 1889)
Gastropoda	Buccinidae	*Chauvetia brunnea*	(Donovan, 1804)
Gastropoda	Buccinidae	*Chauvetia lefebvrii*	(Maravigna, 1840)
Gastropoda	Buccinidae	*Chauvetia mamillata*	(Risso, 1826)
Gastropoda	Buccinidae	*Chauvetia recondita*	(Brugnone, 1873)
Gastropoda	Buccinidae	*Chauvetia mamillata*	(Risso, 1826)
Gastropoda	Buccinidae	*Chauvetia turritellata*	(Deshayes, 1835)
Gastropoda	Buccinidae	*Enginella leucozona*	(Philippi, 1844)
Gastropoda	Buccinidae	*Euthria cornea*	(Linnaeus, 1758)
Gastropoda	Buccinidae	*Pisania striata*	(Gmelin, 1791)
Gastropoda	Buccinidae	*Pollia* sp.	
Gastropoda	Buccinidae	*Pollia dorbignyi*	(Payraudeau, 1826)
Gastropoda	Buccinidae	*Pollia scabra*	Locard, 1892
Gastropoda	Buccinidae	*Pollia scacchiana*	(Philippi, 1844)
Gastropoda	Colubrariidae	*Cumia reticulata*	(Blainville, 1829)
Gastropoda	Nassariidae	*Nassarius* sp.	
Gastropoda	Nassariidae	*Nassarius corniculum*	(Olivi, 1792)
Gastropoda	Nassariidae	*Nassarius crenulatus*	(Bruguière, 1792)
Gastropoda	Nassariidae	*Nassarius cuvierii*	(Payraudeau, 1826)
Gastropoda	Nassariidae	*Nassarius incrassatus*	(Strøm, 1768)
Gastropoda	Nassariidae	*Nassarius lima*	(Dillwyn, 1817)
Gastropoda	Nassariidae	*Nassarius mutabilis*	(Linnaeus, 1758)
Gastropoda	Nassariidae	*Nassarius pygmaeus*	(Lamarck, 1822)
Gastropoda	Nassariidae	*Nassarius reticulatus*	(Linnaeus, 1758)
Gastropoda	Nassariidae	*Nassarius unifasciatus*	(Kiener, 1834)
Gastropoda	Columbellidae	*Columbella rustica*	(Linnaeus, 1758)
Gastropoda	Columbellidae	*Mitrella bruggeni*	van Aartsen, Menkhorst & Gittenberger, 1984
Gastropoda	Columbellidae	*Mitrella gervillii*	(Payraudeau, 1826)
Gastropoda	Columbellidae	*Mitrella psilla*	(Duclos, 1846)
Gastropoda	Columbellidae	*Mitrella scripta*	(Linnaeus, 1758)
Gastropoda	Columbellidae	*Mitrella spelta*	(Kobelt, 1889)
Gastropoda	Fasciolariidae	*Aptyxis syracusanus*	(Linnaeus, 1758)
Gastropoda	Fasciolariidae	*Tarantinaea lignaria*	(Linnaeus, 1758)
Gastropoda	Fasciolariidae	*Fusinus* sp.	
Gastropoda	Fasciolariidae	*Fusinus pulchellus*	(Philippi, 1840)
Gastropoda	Fasciolariidae	*Fusinus rostratus*	(Olivi, 1792)
Gastropoda	Fasciolariidae	*Fusinus rudis*	(Philippi, 1844)
Gastropoda	Fasciolariidae	*Aptyxis syracusanus*	(Linnaeus, 1758)
Gastropoda	Fasciolariidae	*Tarantinaea lignaria*	(Linnaeus, 1758)
Gastropoda	Conidae	*Conus ventricosus*	Gmelin, 1791
Gastropoda	Drilliidae	*Crassopleura maravignae*	(Bivona Ant. in Bivona And., 1838)
Gastropoda	Horaiclavidae	*Haedropleura septangularis*	(Montagu, 1803)
Gastropoda	Clavatulidae	*Fusiturris undatiruga*	(Bivona Ant. in Bivona And., 1838)
Gastropoda	Clathurellidae	*Clathromangelia granum*	(Philippi, 1844)
Gastropoda	Clathurellidae	*Clathromangelia quadrillum*	(Dujardin, 1837)
Gastropoda	Clathurellidae	*Comarmondia gracilis*	(Montagu, 1803)
Gastropoda	Mitromorphidae	*Mitromorpha* sp.	
Gastropoda	Mitromorphidae	*Mitromorpha columbellaria*	(Scacchi, 1836)
Gastropoda	Mitromorphidae	*Mitromorpha crenipicta*	(Dautzenberg, 1889)
Gastropoda	Mitromorphidae	*Mitromorpha olivoidea*	(Cantraine, 1835)
Gastropoda	Mitromorphidae	*Mitromorpha wilhelminae*	(van Aartsen, Menkhorst & Gittenberger, 1984)
Gastropoda	Mangeliidae	*Bela zenetouae*	(van Aartsen, 1988)
Gastropoda	Mangeliidae	*Bela zonata*	(Locard, 1892)
Gastropoda	Mangeliidae	*Mangelia* sp.	
Gastropoda	Mangeliidae	*Mangelia attenuata*	(Montagu, 1803)
Gastropoda	Mangeliidae	*Mangelia multilineolata*	(Deshayes, 1835)
Gastropoda	Mangeliidae	*Mangelia paciniana*	(Calcara, 1839)
Gastropoda	Mangeliidae	*Mangelia stosiciana*	Brusina, 1869
Gastropoda	Mangeliidae	*Mangelia taeniata*	(Deshayes, 1835)
Gastropoda	Mangeliidae	*Mangelia unifasciata*	(Deshayes, 1835)
Gastropoda	Mangeliidae	*Mangelia vauquelini*	(Payraudeau, 1826)
Gastropoda	Mangeliidae	*Sorgenfreispira brachystoma*	(Philippi, 1844)
Gastropoda	Raphitomidae	*Raphitoma mirabilis*	(Pallary, 1904)
Gastropoda	Raphitomidae	*Raphitoma* sp.	
Gastropoda	Raphitomidae	*Raphitoma bicolor*	(Risso, 1826)
Gastropoda	Raphitomidae	*Raphitoma concinna*	(Scacchi, 1836)
Gastropoda	Raphitomidae	*Raphitoma densa*	(Monterosato, 1884)
Gastropoda	Raphitomidae	*Raphitoma echinata*	(Brocchi, 1814)
Gastropoda	Raphitomidae	*Raphitoma horrida*	(Monterosato, 1884)
Gastropoda	Raphitomidae	*Raphitoma laviae*	(Philippi, 1844)
Gastropoda	Raphitomidae	*Raphitoma leufroyi*	(Michaud, 1828)
Gastropoda	Raphitomidae	*Raphitoma linearis*	(Montagu, 1803)
Gastropoda	Raphitomidae	*Raphitoma lineolata*	(Bucquoy, Dautzenberg & Dollfus, 1883)
Gastropoda	Raphitomidae	*Raphitoma philberti*	(Michaud, 1829)
Gastropoda	Raphitomidae	*Raphitoma purpurea*	(Montagu, 1803)
Gastropoda	Architectonicidae	*Pseudotorinia architae*	(O. G. Costa, 1841)
Gastropoda	Rissoellidae	*Rissoella diaphana*	(Alder, 1848)
Gastropoda	Rissoellidae	*Rissoella inflata*	(Monterosato, 1880)
Gastropoda	Rissoellidae	*Rissoella opalina*	(Jeffreys, 1848)
Gastropoda	Rissoinidae	*Rissoina bruguieri*	(Payraudeau, 1826)
Gastropoda	Omalogyridae	*Ammonicera fischeriana*	(Monterosato, 1869)
Gastropoda	Omalogyridae	*Ammonicera rota*	(Forbes & Hanley, 1850)
Gastropoda	Omalogyridae	*Omalogyra atomus*	(Philippi, 1841)
Gastropoda	Omalogyridae	*Omalogyra simplex*	(Costa O. G., 1861)
Gastropoda	Pyramidellidae	*Auristomia erjaveciana*	(Brusina, 1869)
Gastropoda	Pyramidellidae	*Brachystomia eulimoides*	(Hanley, 1844)
Gastropoda	Pyramidellidae	*Eulimella acicula*	(Philippi, 1836)
Gastropoda	Pyramidellidae	*Euparthenia humboldti*	(Risso, 1826)
Gastropoda	Pyramidellidae	*Folinella excavata*	(Phillippi, 1836)
Gastropoda	Pyramidellidae	*Jordaniella nivosa*	(Montagu, 1803)
Gastropoda	Pyramidellidae	*Megastomia conoidea*	(Brocchi, 1814)
Gastropoda	Pyramidellidae	*Noemiamea dolioliformis*	(Jeffreys, 1848)
Gastropoda	Pyramidellidae	*Odostomella doliolum*	(Philippi, 1844)
Gastropoda	Pyramidellidae	*Odostomia* sp.	
Gastropoda	Pyramidellidae	*Odostomia acuta*	Jeffreys, 1848
Gastropoda	Pyramidellidae	*Megastomia conoidea*	(Brocchi, 1814)
Gastropoda	Pyramidellidae	*Odostomia kromi*	van Aartsen, Menkhorst & Gittenberger, 1984
Gastropoda	Pyramidellidae	*Odostomia lukisii*	Jeffreys, 1859
Gastropoda	Pyramidellidae	*Odostomia plicata*	(Montagu, 1803)
Gastropoda	Pyramidellidae	*Brachystomia scalaris*	(MacGillivray, 1843)
Gastropoda	Pyramidellidae	*Odostomia striolata*	Forbes & Hanley, 1850
Gastropoda	Pyramidellidae	*Ondina vitrea*	(Brusina, 1866)
Gastropoda	Pyramidellidae	*Ondina warreni*	(Thompson, 1845)
Gastropoda	Pyramidellidae	*Parthenina emaciata*	(Brusina, 1866)
Gastropoda	Pyramidellidae	*Parthenina indistincta*	(Montagu, 1808)
Gastropoda	Pyramidellidae	*Parthenina interstincta*	(J. Adams, 1797)
Gastropoda	Pyramidellidae	*Parthenina juliae*	(de Folin, 1872)
Gastropoda	Pyramidellidae	*Parthenina monozona*	(Brusina, 1869)
Gastropoda	Pyramidellidae	*Parthenina terebellum*	(Philippi, 1844)
Gastropoda	Pyramidellidae	*Pyrgiscus crenatus*	(Brown, 1827)
Gastropoda	Pyramidellidae	*Pyrgiscus jeffreysii*	(Jeffreys, 1848)
Gastropoda	Pyramidellidae	*Pyrgiscus rufus*	(Philippi, 1836)
Gastropoda	Pyramidellidae	*Spiralinella incerta*	(Milaschewich, 1916)
Gastropoda	Pyramidellidae	*Strioturbonilla sigmoidea*	(Monterosato, 1880)
Gastropoda	Pyramidellidae	*Tragula fenestrata*	(Jeffreys, 1848)
Gastropoda	Pyramidellidae	*Turbonilla acuta*	(Donovan, 1804)
Gastropoda	Pyramidellidae	*Turbonilla gradata*	Bucquoy, Dautzenberg & Dollfus, 1883
Gastropoda	Pyramidellidae	*Turbonilla lactea*	(Linnaeus, 1758)
Gastropoda	Pyramidellidae	*Turbonilla pulchella*	(d'Orbigny, 1841)
Gastropoda	Pyramidellidae	*Turbonilla pumila*	Seguenza G., 1876
Gastropoda	Pyramidellidae	*Turbonilla pusilla*	(Philippi, 1844)
Gastropoda	Pyramidellidae	*Turbonilla sinuosa*	(Jeffreys, 1884)
Gastropoda	Amathinidae	*Clathrella clathrata*	(Philippi, 1844)
Gastropoda	Murchisonellidae	*Ebala pointeli*	(de Folin, 1868)
Gastropoda	Tofanellidae	*Graphis albida*	(Kanmacher, 1798)
Gastropoda	Acteonidae	*Acteon tornatilis*	(Linnaeus, 1758)
Gastropoda	Ringiculidae	*Ringicula auriculata*	(Ménard de la Groye, 1811)
Gastropoda	Ringiculidae	*Ringicula conformis*	Monterosato, 1877
Gastropoda	Bullidae	*Bulla striata*	Bruguière, 1792
Gastropoda	Haminoeidae	*Atys jeffreysi*	(Weinkauff, 1866)
Gastropoda	Haminoeidae	*Haminoea exigua*	Schaefer, 1992
Gastropoda	Haminoeidae	*Haminoea hydatis*	(Linnaeus, 1758)
Gastropoda	Haminoeidae	*Haminoea navicula*	(da Costa, 1778)
Gastropoda	Haminoeidae	*Haminoea orbignyana*	(Férussac, 1822)
Gastropoda	Haminoeidae	*Weinkauffia turgidula*	(Forbes, 1844)
Gastropoda	Philinidae	*Philine catena*	(Montagu, 1803)
Gastropoda	Philinidae	*Philine quadripartita*	Ascanius, 1772
Gastropoda	Aglajidae	*Aglaja tricolorata*	Renier, 1807
Gastropoda	Aglajidae	*Chelidonura africana*	Pruvot-Fol, 1953
Gastropoda	Cylichnidae	*Cylichna cylindracea*	(Pennant, 1777)
Gastropoda	Retusidae	*Pyrunculus hoernesii*	(Weinkauff, 1866)
Gastropoda	Retusidae	*Retusa candidula*	(Locard, 1892)
Gastropoda	Retusidae	*Retusa laevisculpta*	(Granata-Grillo, 1877)
Gastropoda	Retusidae	*Retusa minutissima*	(Monterosato, 1878)
Gastropoda	Retusidae	*Retusa truncatula*	(Bruguière, 1792)
Gastropoda	Retusidae	*Retusa umbilicata*	(Montagu, 1803)
Gastropoda	Rhizoridae	*Volvulella acuminata*	(Bruguière, 1792)
Gastropoda	Runcinidae	*Runcina* sp.	
Gastropoda	Runcinidae	*Runcina coronata*	(Quatrefages, 1844)
Gastropoda	Runcinidae	*Runcina ferruginea*	Kress, 1977
Gastropoda	Oxynoidae	*Lobiger serradifalci*	(Calcara, 1840)
Gastropoda	Oxynoidae	*Oxynoe olivacea*	Rafinesque, 1814
Gastropoda	Plakobranchidae	*Elysia timida*	(Risso, 1818)
Gastropoda	Plakobranchidae	*Elysia viridis*	(Montagu, 1804)
Gastropoda	Plakobranchidae	*Thuridilla hopei*	(Vérany, 1853)
Gastropoda	Limapontiidae	*Limapontia capitata*	(O. F. Müller, 1774)
Gastropoda	Limapontiidae	*Placida verticilata*	Ortea, 1982
Gastropoda	Umbraculidae	*Umbraculum umbraculum*	(Lightfoot, 1786)
Gastropoda	Tylodinidae	*Tylodina perversa*	(Gmelin, 1791)
Gastropoda	Aplysiidae	*Aplysia* sp.	
Gastropoda	Aplysiidae	*Aplysia depilans*	Gmelin, 1791
Gastropoda	Aplysiidae	*Aplysia fasciata*	Poiret, 1789
Gastropoda	Aplysiidae	*Aplysia parvula*	Mörch, 1863
Gastropoda	Aplysiidae	*Aplysia punctata*	(Cuvier, 1803)
Gastropoda	Aplysiidae	*Petalifera petalifera*	(Rang, 1828)
Gastropoda	Aplysiidae	*Phyllaplysia lafonti*	P. Fischer, 1872
Gastropoda	Pleurobranchidae	*Berthella aurantiaca*	(Risso, 1818)
Gastropoda	Pleurobranchidae	*Pleurobranchus membranaceus*	(Montagu, 1816)
Gastropoda	Discodorididae	*Paradoris indecora*	(Bergh, 1881)
Gastropoda	Discodorididae	*Peltodoris atromaculata*	Bergh, 1880
Gastropoda	Discodorididae	*Tayuva lilacina*	(Gould, 1852)
Gastropoda	Chromodorididae	*Felimare orsinii*	(Vérany, 1846)
Gastropoda	Chromodorididae	*Felimare picta*	(Schultz in Philippi, 1836)
Gastropoda	Chromodorididae	*Felimare tricolor*	(Cantraine, 1835)
Gastropoda	Chromodorididae	*Felimare villafranca*	(Risso, 1818)
Gastropoda	Chromodorididae	*Felimida krohni*	(Vérany, 1846)
Gastropoda	Phyllidiidae	*Phyllidia flava*	Aradas, 1847
Gastropoda	Dendrodorididae	*Dendrodoris limbata*	(Cuvier, 1804)
Gastropoda	Dendrodorididae	*Dendrodoris grandiflora*	(Rapp, 1827)
Gastropoda	Dendrodorididae	*Doriopsilla areolata*	Bergh, 1880
Gastropoda	Onchidorididae	*Onchidoris neapolitana*	(Delle Chiaje, 1841)
Bivalvia	Tellinidae	*Gastrana fragilis*	(Linnaeus, 1758)
Bivalvia	Tellinidae	*Macoma cumana*	(O. G. Costa, 1830)
Bivalvia	Tellinidae	*Atlantella distorta*	(Poli, 1791)
Bivalvia	Tellinidae	*Tellina albicans*	Gmelin, 1791
Bivalvia	Tellinidae	*Tellina fabula*	Gmelin, 1791
Bivalvia	Tellinidae	*Tellina planata*	Linnaeus, 1758
Bivalvia	Tellinidae	*Tellina pulchella*	Lamarck, 1818
Bivalvia	Tellinidae	*Tellina tenuis*	da Costa, 1778
Gastropoda	Goniodorididae	*Goniodoris castanea*	Alder & Hancock, 1845
Gastropoda	Goniodorididae	*Trapania maculata*	Haefelfinger, 1960
Gastropoda	Polyceridae	*Limacia clavigera*	(O. F. Müller, 1776)
Gastropoda	Polyceridae	*Palio dubia*	(M. Sars, 1829)
Gastropoda	Polyceridae	*Polycera quadrilineata*	(O. F. Müller, 1776)
Gastropoda	Polyceridae	*Polycerella emertoni*	A. E. Verrill, 1880
Gastropoda	Aegiridae	*Aegires leuckartii*	Vérany, 1853
Gastropoda	Tritoniidae	*Tritonia lineata*	Alder & Hancock, 1848
Gastropoda	Tritoniidae	*Tritonia manicata*	Deshayes, 1853
Gastropoda	Proctonotidae	*Janolus cristatus*	(Delle Chiaje, 1841)
Gastropoda	Facelinidae	*Caloria elegans*	(Alder & Hancock, 1845)
Gastropoda	Facelinidae	*Cratena peregrina*	(Gmelin, 1791)
Gastropoda	Facelinidae	*Favorinus branchialis*	(Rathke, 1806)
Gastropoda	Flabellinidae	*Calmella cavolini*	(Vérany, 1846)
Gastropoda	Flabellinidae	*Flabellina affinis*	(Gmelin, 1791)
Gastropoda	Flabellinidae	*Flabellina lineata*	(Lovén, 1846)
Gastropoda	Flabellinidae	*Flabellina pedata*	(Montagu, 1816)
Gastropoda	Calmidae	*Calma glaucoides*	(Alder & Hancock, 1854)
Gastropoda	Tergipedidae	*Cuthona caerulea*	(Montagu, 1804)
Gastropoda	Tergipedidae	*Cuthona genovae*	(O'Donoghue, 1929)
Gastropoda	Tergipedidae	*Tergipes tergipes*	(Forsskål in Niebuhr, 1775)
Gastropoda	Siphonariidae	*Williamia gussoni*	(Costa O. G., 1829)
Gastropoda	Ellobiidae	*Trimusculus mammillaris*	(Linnaeus, 1758)
Bivalvia	Nuculidae	*Nucula nitidosa*	Winckworth, 1930
Bivalvia	Nuculanidae	*Lembulus pella*	(Linnaeus, 1767)
Bivalvia	Arcidae	*Acar clathrata*	(Defrance, 1816)
Bivalvia	Arcidae	*Anadara corbuloides*	(Monterosato, 1878)
Bivalvia	Arcidae	*Arca noae*	Linnaeus, 1758
Bivalvia	Arcidae	*Arca tetragona*	Poli, 1795
Bivalvia	Arcidae	*Asperarca nodulosa*	(O. F. Müller, 1776)
Bivalvia	Arcidae	*Barbatia barbata*	(Linnaeus, 1758)
Bivalvia	Noetiidae	*Striarca lactea*	(Linnaeus, 1758)
Bivalvia	Glycymerididae	*Glycymeris nummaria*	(Linnaeus, 1758)
Bivalvia	Mytilidae	*Musculus subpictus*	(Cantraine, 1835)
Bivalvia	Mytilidae	*Amygdalum politum*	(Verrill & S. Smith [in Verrill], 1880)
Bivalvia	Mytilidae	*Arcuatula senhousia*	(Benson in Cantor, 1842)
Bivalvia	Mytilidae	*Brachidontes pharaonis*	(P. Fischer, 1870)
Bivalvia	Mytilidae	*Crenella pellucida*	(Jeffreys, 1859)
Bivalvia	Mytilidae	*Gibbomodiola adriatica*	(Lamarck, 1819)
Bivalvia	Mytilidae	*Gregariella petagnae*	(Scacchi, 1832)
Bivalvia	Mytilidae	*Jolya martorelli*	(Hidalgo, 1878)
Bivalvia	Mytilidae	*Lithophaga lithophaga*	(Linnaeus, 1758)
Bivalvia	Mytilidae	*Modiolula phaseolina*	(Philippi, 1844)
Bivalvia	Mytilidae	*Modiolus barbatus*	(Linnaeus, 1758)
Bivalvia	Mytilidae	*Musculus costulatus*	(Risso, 1826)
Bivalvia	Mytilidae	*Musculus discors*	(Linnaeus, 1767)
Bivalvia	Mytilidae	*Musculus niger*	(J.E. Gray, 1824)
Bivalvia	Mytilidae	*Mytilaster lineatus*	(Gmelin, 1791)
Bivalvia	Mytilidae	*Mytilaster minimus*	(Poli, 1795)
Bivalvia	Mytilidae	*Mytilus* sp.	
Bivalvia	Mytilidae	*Mytilus galloprovincialis*	Lamarck, 1819
Bivalvia	Mytilidae	*Rhomboidella prideauxi*	(Leach, 1815)
Bivalvia	Mytilidae	*Mytilus edulis*	Linnaeus, 1758
Bivalvia	Pinnidae	*Pinna nobilis*	Linnaeus, 1758
Bivalvia	Pteriidae	*Pinctada imbricata radiata*	(Leach, 1814)
Bivalvia	Pectinidae	*Aequipecten opercularis*	(Linnaeus, 1758)
Bivalvia	Pectinidae	*Flexopecten glaber*	(Linnaeus, 1758)
Bivalvia	Pectinidae	*Flexopecten hyalinus*	(Poli, 1795)
Bivalvia	Pectinidae	*Manupecten pesfelis*	(Linnaeus, 1758)
Bivalvia	Pectinidae	*Mimachlamys varia*	(Linnaeus, 1758)
Bivalvia	Pectinidae	*Pecten* sp.	
Bivalvia	Pectinidae	*Talochlamys multistriata*	(Poli, 1795)
Bivalvia	Spondylidae	*Spondylus gaederopus*	Linnaeus, 1758
Bivalvia	Anomiidae	*Anomia ephippium*	Linnaeus, 1758
Bivalvia	Limidae	*Lima lima*	(Linnaeus, 1758)
Bivalvia	Limidae	*Limaria hians*	(Gmelin, 1791)
Bivalvia	Limidae	*Limaria loscombi*	(G. B. Sowerby I, 1823)
Bivalvia	Limidae	*Limaria tuberculata*	(Olivi, 1792)
Bivalvia	Limidae	*Limatula subovata*	(Monterosato, 1875)
Bivalvia	Limidae	*Limaria hians*	(Gmelin, 1791)
Bivalvia	Ostreidae	*Crassostrea gigas*	(Thunberg, 1793)
Bivalvia	Ostreidae	*Ostrea edulis*	Linnaeus, 1758
Bivalvia	Ostreidae	*Ostrea stentina*	Payraudeau, 1826
Bivalvia	Gryphaeidae	*Neopycnodonte cochlear*	(Poli, 1795)
Bivalvia	Carditidae	*Cardita calyculata*	(Linnaeus, 1758)
Bivalvia	Carditidae	*Cardites antiquatus*	(Linnaeus, 1758)
Bivalvia	Carditidae	*Centrocardita aculeata*	(Poli, 1795)
Bivalvia	Carditidae	*Glans trapezia*	(Linnaeus, 1767)
Bivalvia	Cardiidae	*Acanthocardia aculeata*	(Linnaeus, 1758)
Bivalvia	Cardiidae	*Acanthocardia paucicostata*	(G. B. Sowerby II, 1834)
Bivalvia	Cardiidae	*Acanthocardia tuberculata*	(Linnaeus, 1758)
Bivalvia	Cardiidae	*Fulvia fragilis*	(Forsskål in Niebuhr, 1775)
Bivalvia	Cardiidae	*Papillicardium papillosum*	(Poli, 1791)
Bivalvia	Cardiidae	*Parvicardium* sp.	
Bivalvia	Cardiidae	*Parvicardium exiguum*	(Gmelin, 1791)
Bivalvia	Cardiidae	*Parvicardium minimum*	(Philippi, 1836)
Bivalvia	Cardiidae	*Parvicardium pinnulatum*	(Conrad, 1831)
Bivalvia	Cardiidae	*Parvicardium scriptum*	(Bucquoy, Dautzenberg & Dollfus, 1892)
Bivalvia	Cardiidae	*Parvicardium vroomi*	van Aartsen, Menkhorst & Gittenberger, 1984
Bivalvia	Lucinidae	*Ctena decussata*	(O. G. Costa, 1829)
Bivalvia	Lucinidae	*Loripes lucinalis*	(Lamarck, 1818)
Bivalvia	Lucinidae	*Loripinus fragilis*	(Philippi, 1836)
Bivalvia	Lucinidae	*Lucinella divaricata*	(Linnaeus, 1758)
Bivalvia	Lucinidae	*Myrtea spinifera*	(Montagu, 1803)
Bivalvia	Thyasiridae	*Thyasira flexuosa*	(Montagu, 1803)
Bivalvia	Chamidae	*Chama asperella*	Lamarck, 1819
Bivalvia	Chamidae	*Chama gryphoides*	Linnaeus, 1758
Bivalvia	Chamidae	*Pseudochama gryphina*	(Lamarck, 1819)
Bivalvia	Galeommatidae	*Galeomma turtoni*	Turton, 1825
Bivalvia	Kelliidae	*Kellia suborbicularis*	(Montagu, 1803)
Bivalvia	Lasaeidae	*Hemilepton nitidum*	(Turton, 1822)
Bivalvia	Lasaeidae	*Lasaea adansoni*	(Gmelin, 1791)
Bivalvia	Lasaeidae	*Scacchia oblonga*	(Philippi, 1836)
Bivalvia	Lasaeidae	*Lasaea adansoni*	(Gmelin, 1791)
Bivalvia	Montacutidae	*Kurtiella bidentata*	(Montagu, 1803)
Bivalvia	Mactridae	*Eastonia rugosa*	(Helbling, 1779)
Bivalvia	Mactridae	*Mactra stultorum*	(Linnaeus, 1758)
Bivalvia	Mactridae	*Spisula subtruncata*	(da Costa, 1778)
Bivalvia	Donacidae	*Donax semistriatus*	Poli, 1795
Bivalvia	Donacidae	*Donax venustus*	Poli, 1795
Bivalvia	Psammobiidae	*Gari depressa*	(Pennant, 1777)
Bivalvia	Semelidae	*Abra* sp.	
Bivalvia	Semelidae	*Abra alba*	(W. Wood, 1802)
Bivalvia	Semelidae	*Abra nitida*	(O. F. Müller, 1776)
Bivalvia	Semelidae	*Scrobicularia cottardii*	(Payraudeau, 1826)
Bivalvia	Solecurtidae	*Azorinus chamasolen*	(da Costa, 1778)
Bivalvia	Trapezidae	*Coralliophaga lithophagella*	(Lamarck, 1819)
Bivalvia	Veneridae	*Chamelea gallina*	(Linnaeus, 1758)
Bivalvia	Veneridae	*Clausinella fasciata*	(da Costa, 1778)
Bivalvia	Veneridae	*Dosinia exoleta*	(Linnaeus, 1758)
Bivalvia	Veneridae	*Dosinia lupinus*	(Linnaeus, 1758)
Bivalvia	Veneridae	*Gouldia minima*	(Montagu, 1803)
Bivalvia	Veneridae	*Irus irus*	(Linnaeus, 1758)
Bivalvia	Veneridae	*Lajonkairia lajonkairii*	(Payraudeau, 1826)
Bivalvia	Veneridae	*Petricola lithophaga*	(Retzius, 1788)
Bivalvia	Veneridae	*Petricola substriata*	Montagu, 1808
Bivalvia	Veneridae	*Pitar rudis*	(Poli, 1795)
Bivalvia	Veneridae	*Polititapes aureus*	(Gmelin, 1791)
Bivalvia	Veneridae	*Polititapes rhomboides*	(Pennant, 1777)
Bivalvia	Veneridae	*Ruditapes decussatus*	(Linnaeus, 1758)
Bivalvia	Veneridae	*Venerupis corrugata*	(Gmelin, 1791)
Bivalvia	Veneridae	*Venerupis geographica*	(Gmelin, 1791)
Bivalvia	Veneridae	*Venus verrucosa*	Linnaeus, 1758
Bivalvia	Neoleptonidae	*Neolepton sulcatulum*	(Jeffreys, 1859)
Bivalvia	Myidae	*Sphenia binghami*	Turton, 1822
Bivalvia	Corbulidae	*Corbula gibba*	(Olivi, 1792)
Bivalvia	Corbulidae	*Lentidium mediterraneum*	(O. G. Costa, 1830)
Bivalvia	Gastrochaenidae	*Rocellaria dubia*	(Pennant, 1777)
Bivalvia	Solenidae	*Solen marginatus*	Pulteney, 1799
Bivalvia	Pharidae	*Pharus legumen*	(Linnaeus, 1758)
Bivalvia	Pharidae	*Phaxas pellucidus*	(Pennant, 1777)
Bivalvia	Hiatellidae	*Hiatella arctica*	(Linnaeus, 1767)
Bivalvia	Hiatellidae	*Hiatella rugosa*	(Linnaeus, 1767)
Bivalvia	Thraciidae	*Thracia distorta*	(Montagu, 1803)
Bivalvia	Clavagellidae	*Bryopa aperta*	(G. B. Sowerby I, 1823)
Bivalvia	Pandoridae	*Pandora inaequivalvis*	(Linnaeus, 1758)
Scaphopoda	Dentaliidae	*Antalis novemcostata*	(Lamarck, 1818)
Scaphopoda	Dentaliidae	*Antalis vulgaris*	(da Costa, 1778)
Scaphopoda	Fustiariidae	*Fustiaria rubescens*	(Deshayes, 1825)
Cephalopoda	Sepiidae	*Sepia officinalis*	Linnaeus, 1758
Cephalopoda	Octopodidae	*Octopus vulgaris*	Cuvier, 1797
